# Mitochondria Transplantation to Bone Marrow Stromal Cells Promotes Angiogenesis During Bone Repair

**DOI:** 10.1002/advs.202403201

**Published:** 2024-08-13

**Authors:** Yifan Wang, Wenjing Li, Yusi Guo, Ying Huang, Yaru Guo, Jia Song, Feng Mei, Peiwen Liao, Zijian Gong, Xiaopei Chi, Xuliang Deng

**Affiliations:** ^1^ National Engineering Laboratory for Digital and Material Technology of Stomatology NMPA Key Laboratory for Dental Materials & Beijing Laboratory of Biomedical Materials Department of Geriatric Dentistry Peking University School and Hospital of Stomatology Beijing 100081 P. R. China; ^2^ National Engineering Laboratory for Digital and Material Technology of Stomatology NMPA Key Laboratory for Dental Materials & Beijing Laboratory of Biomedical Materials Department of Geriatric Dentistry Peking University School and Hospital of Stomatology People's Republic of China. Peking University Health Science Center and Hospital of Stomatology Beijing 100081 P. R. China; ^3^ Department of Stomatology Union Hospital Tongji Medical College Huazhong University of Science and Technology Wuhan 430022 P. R. China; ^4^ Peking University Health Science Center and Hospital of Stomatology Beijing 100081 P. R. China

**Keywords:** angiogenesis, bone marrow stromal cells, bone repair, co‐transplantation, endothelial cells, mitochondria, mitochondria transplantation

## Abstract

Angiogenesis is crucial for successful bone defect repair. Co‐transplanting Bone Marrow Stromal Cells (BMSCs) and Endothelial Cells (ECs) has shown promise for vascular augmentation, but it face challenges in hostile tissue microenvironments, including poor cell survival and limited efficacy. In this study, the mitochondria of human BMSCs are isolated and transplanted to BMSCs from the same batch and passage number (BMSCs^mito^). The transplanted mitochondria significantly boosted the ability of BMSCs^mito^‐ECs to promote angiogenesis, as assessed by in vitro tube formation and spheroid sprouting assays, as well as in vivo transplantation experiments in balb/c mouse and SD rat models. The Dll4‐Notch1 signaling pathway is found to play a key role in BMSCs^mito^‐induced endothelial tube formation. Co‐transplanting BMSCs^mito^ with ECs in a rat cranial bone defect significantly improves functional vascular network formation, and improve bone repair outcomes. These findings thus highlight that mitochondrial transplantation, by acting through the DLL4‐Notch1 signaling pathway, represents a promising therapeutic strategy for enhancing angiogenesis and improving bone repair. Hence, mitochondrial transplantation to BMSCS as a therapeutic approach for promoting angiogenesis offers valuable insights and holds much promise for innovative regenerative medicine therapies.

## Graphical abstract

This study focuses on enhancing angiogenesis for effective bone repair by isolating and transplanting mitochondria from donor to recipient BMSCs (BMSCs^mito^) of the same batch and passage number. The BMSCs^mito^ exhibit significant improvements in pro‐angiogenic functions with various in vitro and in vivo assays, thus highlighting the crucial role of the Dll4‐Notch1 signaling pathway. Co‐transplantation of BMSCs^mito^ with ECs in a rat cranial bone defect model significantly enhances functional vascular network formation, demonstrating the vast potential of mitochondrial transplantation, mediated through the DLL4‐Notch1 signaling pathway, as a promising therapeutic strategy for regenerative medicine.

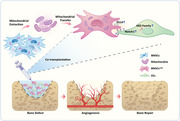

## 1 Introduction

Bone defect repair is a complex process that, while often occurring spontaneously and efficiently, can present significant challenges in certain clinical contexts. A key factor in this process is angiogenesis, which plays a crucial role by supplying osteogenic progenitors, facilitating oxygen transport, nutrient delivery, and metabolic waste removal during bone repair, thereby significantly affecting the success of bone defect healing.^[^ ^1^ ^]^ Conventional therapeutic modalities that facilitate bone defect healing are largely dependent on growth factors targeting endothelial cell activation to initiate angiogenesis.^[^ ^2^ ^]^ However, these approaches frequently fall short of effectively promoting comprehensive angiogenesis due to the complex and multifaceted nature of this biological process.^[^ ^3^ ^]^

Previous studies have shown that BMSCs, well known for their osteogenic potential, can also enhance angiogenesis by activating ECs.^[^ ^4^, ^5^ ^]^ The co‐transplantation of BMSCs and ECs has been widely applied for improving vascular formation and bone repair.^[^ ^4^, ^6^, ^7^, ^8^ ^]^ However, despite their potential in angiogenesis and bone regeneration, transplanted BMSCs and ECs face several challenges in hostile pathological tissue microenvironments, including low survival rates and inadequate cell‐to‐cell interactions.^[^ ^9^ ^]^ These limitations impair the formation of crucial vascular networks necessary for effective bone healing, thereby constraining the ability of BMSCs to achieve sustained bone repair in clinical applications.

Mitochondria, being integral to energy metabolism and essential for various key cellular processes, play a pivotal role in cellular functions. Notably, mitochondrial transplantation is known to boost various cellular functions under various physiological and pathological conditions.^[^ ^10^, ^11^ ^]^ Studies have reported that incorporation of exogenous mitochondria leads to increased neuronal proliferation^[^ ^12^ ^]^ and enhanced astrocyte functionality during stroke.^[^ ^13^ ^]^

Mitochondrial transplantation involves isolating mitochondria from donor cells and transferring them into recipient cells. Our previous research^[^ ^14^ ^]^ confirmed that mitochondrial transplantation improves the BMSCs proliferation, migration, and osteogenic differentiation of BMSCs. Therefore, our research question is whether mitochondria transplantation can enhance BMSCs‐mediated bone regeneration by promoting vascular formation?

In this study, we isolated mitochondria from human BMSCs and transplanted them into recipient human BMSCs from the same batch and passage number (BMSCs^mito^). This process successfully transfers mitochondria into recipient cells, where the majority fuse with the host cell's mitochondrial network, while some of them might undergo degradation. The transplanted mitochondria significantly boosted the ability of BMSCs^mito^‐ECs to promote angiogenesis within in vitro tube formation and spheroid sprouting assays, as well as with in vivo blab/c mouse and SD rat models. This augmented capacity is attributed to the intricate cell‐cell crosstalk facilitated by the DLL4‐Notch1 signaling pathway. The joint transplantation of BMSCs^mito^ and ECs was demonstrated to significantly enhance the formation of functional blood vessels and bone regeneration within rat cranial bone defects.

## 2 Results and Discussion

### 2.1 Characterization of Isolated Mitochondria Transplantation into Recipient BMSCs

**Figure 1:**
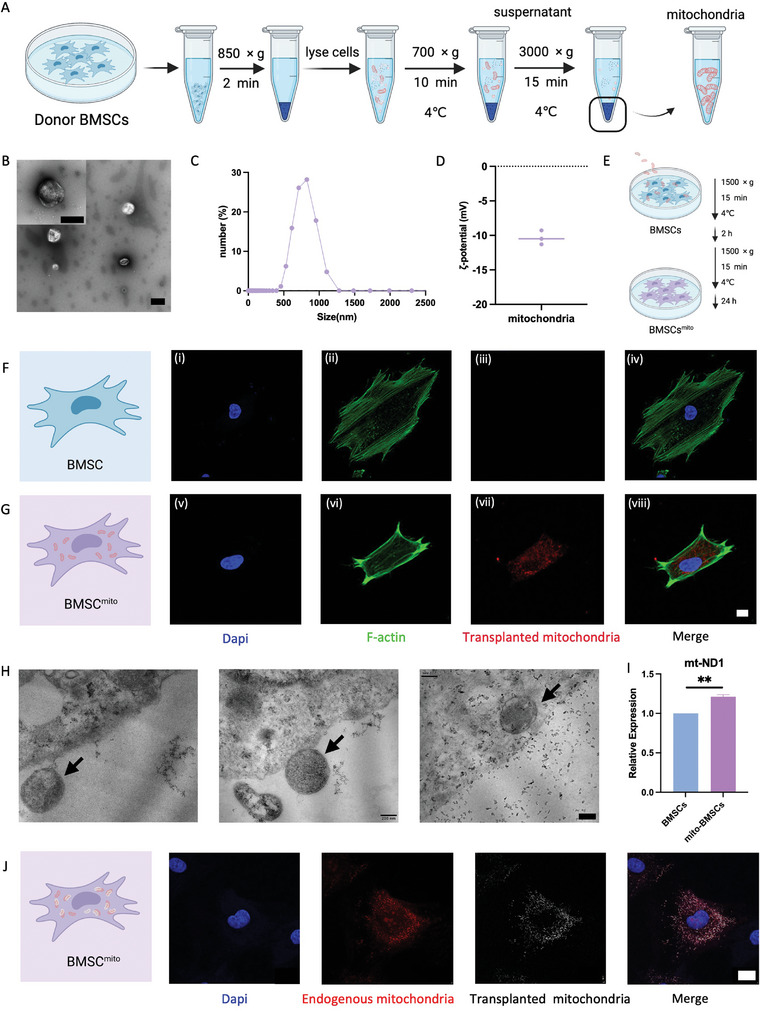
Isolation and characterization of mitochondria and construction of BMSCs^mito^ through mitochondria transplantation into recipient BMSCs. A) Illustration of mitochondria isolation from the donor BMSCs via sequential centrifugation. B) Mitochondrial morphology was detected by transmission electron microscope (TEM). Scale bar, 1 µm. C) Size distribution of mitochondria. D) ζ‐potential values of mitochondria. E) Illustration of mitochondria transplantation into recipient BMSCs by co‐incubation with low‐speed centrifugation. F) BMSC group and G) BMSC^mito^ group: Confocal immunofluorescence staining of cell nuclei with DAPI in BMSCs from the BMSC group (i) and BMSC^mito^ group (v), and F‐actin with FITC‐labeled phalloidin in BMSCs from the BMSC group (ii) and BMSC^mito^ group (vi) at 24 h after mitochondria transplantation. Confocal immunofluorescence staining of transplanted mitochondria labeled before isolation from the BMSC group (iii) and BMSC^mito^ group (vii) at 24 h after mitochondria transplantation. (iv, viii) Merged confocal images of a, b, and c or e, f, and g, respectively. The scale bar represents 20 µm. H) The morphology of free mitochondria as they enter mesenchymal stem cells (MSCs) was observed using TEM. Scale bar: 200 nm. I) The mitochondrial DNA content in BMSCs and BMSC^mito^. J) BMSC^mito^ Group: Confocal immunofluorescence staining DAPI, primordial mitochondria, transplanted mitochondria, and the merged image of the three. Scale bar: 25 µm.

To isolate mitochondria, we utilized a specially‐formulated cell lysis kit that enabled the extraction of intact and biologically active mitochondria via gradient centrifugation from donor BMSCs (**Figure** **1**A). Transmission electron microscopy (TEM) analysis confirmed the presence of intact mitochondria with an approximate diameter of 800 nm, exhibiting a distinctive spherical structure (Figure 1B). Fluid dynamic diameter measurements indicated a mitochondrial diameter of 983.8 ± 219.4 nm (Figure 1C). The ζ‐potential values of mitochondria yielded results of −10.36 ± 1.02 mV. Preserving this structural integrity is crucial, as mitochondria, with their unique morphology and internal organization, contribute to various key cellular functions such as energy production, metabolism, and apoptosis regulation. These analyses thus validate the high purity and structural integrity of the extracted mitochondria.

Subsequently, these isolated mitochondria were transplanted into recipient BMSCs from the same batch and passage number. Employing the Mitoception approach (Figure 1B), the transplantation of isolated mitochondria into recipient BMSCs ensured their integration and functional uptake. To validate the presence of transplanted mitochondria within the recipient BMSCs, we pre‐labeled donor BMSC mitochondria with MitoTracker dye before isolation. Subsequent examination after a 24‐hour period of mitochondria transplantation revealed no detectable fluorescence signals from the donor mitochondria in the control cells (Figure 1F). However, a distinct and discernible fluorescence signal was evident within the recipient cells (Figure 1G). To observe the uptake of free mitochondria by recipient cells, we used TEM at 5, 10, and 15 min post‐mitochondrial transplantation. Our observations confirmed the presence of free mitochondria entering the recipient cells (Figure 1H). This observation unequivocally confirms the successful and precise transplantation of mitochondria into BMSCs, thus corroborating the efficiency and efficacy of our devised mitochondrial transplantation protocol. Studies have shown that the mechanism of mitochondrial transplantation involves entry into the cells through fusion mechanisms, membrane penetration, interactions with the cellular cytoskeleton, formation of intracellular vesicles, and autonomous movement. This process leverages specific interactions with the cell membrane while preserving the integrity and functionality of mitochondria. To investigate the relationship between endogenous and transplanted mitochondria after mitochondrial transplantation, we conducted separate staining of both types in BMSCs. Our staining results revealed significant colocalization of endogenous and transplanted mitochondria within BMSC^mito^. These findings align are consistent with the study of Cowan et al.,^[^ ^15^ ^]^ which similarly reported notable colocalization in their experiments.

### 2.2 Mitochondria‐Promoted Activation of ECs by BMSCs and Enhanced Tube Formation In Vitro

**Figure 2:**
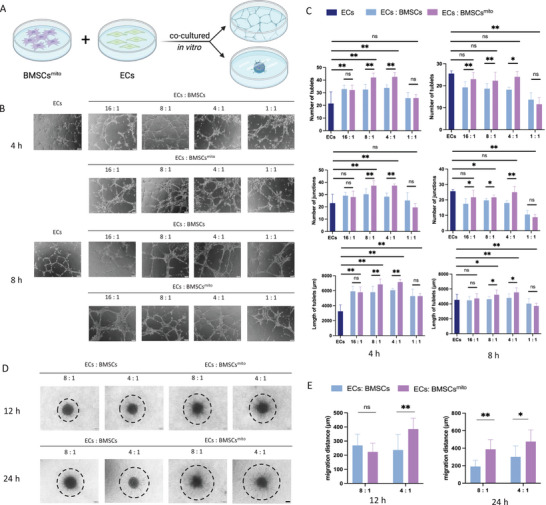
BMSCs^mito^ activated endothelial cells and increased tube formation in vitro. A) Illustration of in vitro angiogenesis assay with co‐culture system. B) Representative images of tube formation at 4 and 8 h after cells were seeded on Matrigel. hUVECs group: Monoculture of human umbilical vein ECs (hUVECs). hUVECs: BMSCs group: hUVECs and human BMSCs (hBMSCs) were co‐cultured at various cell ratios, with the same total number of cells; hUVECs: BMSCs^mito^ group: hUVECs and BMSCs^mito^ were co‐cultured at various cell ratios, with the same total number of cells. Scale bar, 100 µm. C) The total number of tubules, number of junctions, and tube length in each group from Figure A were quantified using the Image Pro Plus software. D) Representative images of spheroid sprouting angiogenesis assay at 12 and 24 h after cell spheroids were seeded on Type I collagen. hUVECs: hBMSCs group: hUVECs and BMSCs were co‐cultured at various cell ratios, with the same total number of cells; hUVECs: BMSCs^mito^ group: hUVECs and BMSCs^mito^ were co‐cultured at various cell ratios, with the same total number of cells. Scale bar, 100 µm. (E) The migration distance in each group from Figure C was quantified using Image Pro Plus software. Results were presented as the mean ± SEM (*n*  =  5). One‐way ANOVA with Tukey's post hoc test was used to determine statistical significance. *p < 0.05, **p < 0.01, ns: no significant difference.

To evaluate the capacity of BMSCs^mito^ in promoting endothelial tube formation, an in vitro tube formation assay was conducted (**Figure** **2**A). ECs were co‐cultured with varying ratios of BMSCs or BMSCs^mito^, ranging from 16:1 to 1:1, and incubated for 4 or 8 h while being observed under a microscope. A dose‐dependent trend was observed with increasing quantities of BMSCs, resulting in a proportional enhancement in the number of tubes, junctions, and tube lengths. Within the in vitro tube formation assay, an increase in the “number of tubes” suggests enhanced angiogenesis, by providing more conduits for delivery of oxygen and nutrients. The “number of junctions” reflects the complexity and stability of the vascular network, while increased “tube lengths” indicate extended vascular structures. These changes collectively signify a more efficient, stable, and extensive vascular network formation. The most optimal angiogenic response was observed at BMSC ratios of 4:1 and 8:1. Importantly, BMSCs^mito^ exhibited significantly enhanced angiogenic potential compared to non‐BMSCs^mito^ and ECs^mito^ at these optimal ratios, a consistent finding at both the 4‐hour and 8‐hour timepoints (Figure 2B,C; Figure S1, Supporting Information).

For a more realistic assessment of the effects of BMSC^mito^ on endothelial and angiogenic capacities, a spheroid sprouting assay was conducted. The outcomes revealed a significant increase in cell sprouting distance and denser branching when ECs were co‐cultured with BMSCs^mito^ at ratios of 4:1 and 8:1 (Figure 2D,E; Figure S1C,D, Supporting Information). With the in vitro spheroid sprouting assay, an increase in migration distance is indicative of enhanced cellular movement away from the spheroid. This signifies improved migratory capacity of cells, which is essential for processes such as angiogenesis. A greater migration distance implies that cells are more effectively reaching out into the surrounding environment, potentially contributing to the formation of new blood vessels or tissue structures. This parameter is crucial for evaluating the pro‐angiogenic potential of the tested conditions within the context of tissue regeneration and repair.

In our experiments, we found that the peak angiogenic capacity occurred at a ratio of 4:1 BMSCs to ECs. This disparity may be attributed to rapid proliferation, potentially enhancing the angiogenic potential of a smaller BMSC population. However, increasing the total number of BMSCs did not proportionally enhance angiogenesis, indirectly suggesting that the observed benefits were not solely due to accelerated BMSC division. We hypothesize that a lower number of BMSCs might limit the beneficial effects of mitochondrial enhancement, while fewer ECs could diminish angiogenic potential.

**Figure 3:**
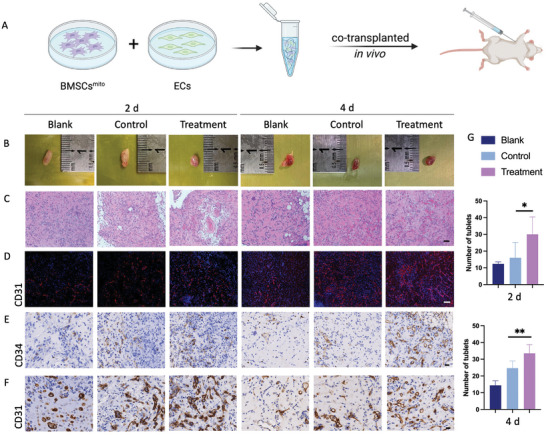
Mitochondria‐facilitated BMSCs co‐transplantation with ECs, enhancing the maturity of the newly formed blood vessels in vivo. Blank group: independent injection of hUVECs. Control group: hUVECs and hBMSCs were co‐cultured at a ratio of 4: 1; Treatment group: hUVECs and BMSCs^mito^ were co‐cultured at a ratio of 4: 1. A) Illustration of the subcutaneous co‐transplantation procedure performed in Balb/c nude mice. B) Photographs of the subcutaneous implants taken at 2 and 4 days post cell injection. Scale bar: 10 mm. C) Representative images of HE staining of the implant tissue sections. Scale bar: 50 µm. D) Immunofluorescence staining for CD31 in the implant tissue sections. Scale bar: 50 µm. E) Immunohistochemical staining for CD34 in the implant tissue sections. Scale bar: 20 µm. F) Immunohistochemical staining for CD31 in the implant tissue sections. Scale bar: 20 µm. G) Quantification of the number of tubules in each group from Figure C using the Image Pro Plus software. Results are presented as the mean ± SEM (*n*  =  5). One‐way ANOVA with Tukey's post hoc test was used to determine statistical significance. *p < 0.05, **p < 0.01.

For further investigations, we evaluated the angiogenic potential of BMSCs^mito^ in stimulating ECs angiogenesis using a subcutaneous tissue culture model in nude mice in vivo. Visual inspection of the subcutaneous tissue revealed a significant increase in newly formed branching blood vessels within the experimental group compared to the control group at 2 and 4 days after surgery (**Figure** **3**A). Histological analyses, incorporating H&E staining and CD31 immunocytochemical staining, depicted an early initiation of blood vessel formation within the experimental group on day 2, which contrasted sharply with the restricted effects observed in the control and blank groups. By day 4, all groups exhibited blood vessel formation, with the experimental group manifesting a significantly better effect (Figure 3B–E; Figure S2, Supporting Information). Studies by Levoux et al.^[^ ^16^ ^]^ and Feng et al.^[^ ^17^ ^]^ also demonstrated the potential of mitochondrial transplantation in stimulating ECs angiogenesis in wound healing, muscle repair, and ischemic conditions. These findings thus collectively validated the potent effects of mitochondrial transplantation in promoting BMSCs to enhance endothelial tube formation.

### 2.3 Direct Cell‐Cell Interaction Facilitated by BMSCs^mito^ Enhanced Angiogenesis

**Figure 4:**
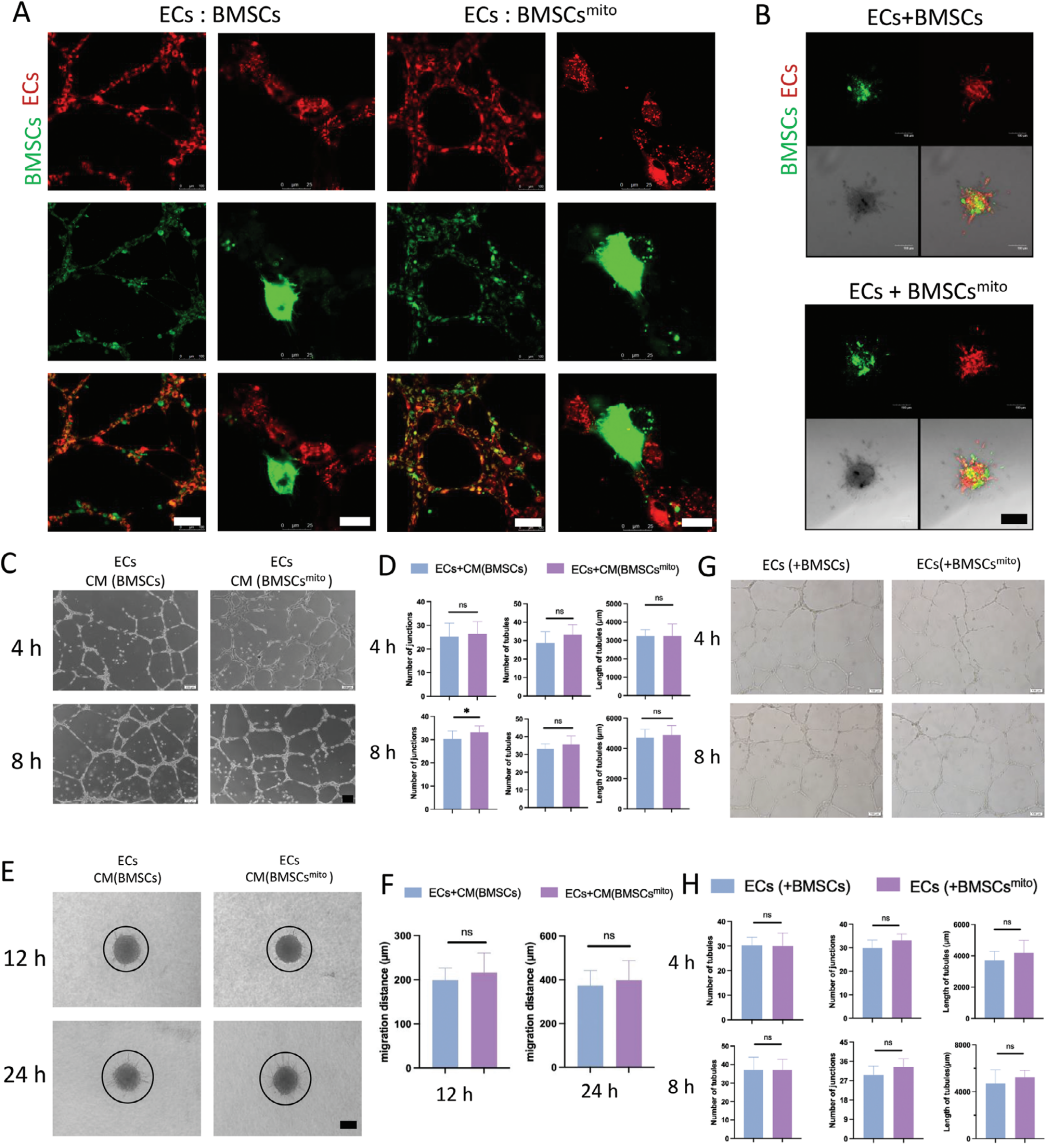
Effects of BMSCs^mito^ stimulation of ECs through direct cell‐to‐cell contact. A) Representative images showing ECs and BMSCs during the tube formation assay. Scale bar: 100 µm. For the higher magnification views, the scale bar: 25 µm. B) Representative images from the spheroid sprouting angiogenesis assay for ECs and BMSCs, highlighting the sprouting structures indicative of angiogenic potential. Scale bar: 100 µm. C) Representative images of in vitro tube formation assay at 4 and 8 h after cells were seeded on Matrigel. EC‐CM(BMSCs) group: hUVECs were cultured in a conditioned medium derived from hBMSCs; EC‐CM(BMSCs^mito^) group: hUVECs were cultured in a conditioned medium derived from hBMSCs^mito^. Scale bar, 100 µm. D) The total number of tubules, number of junctions, and tube lengths in each group in Figure C were quantified using the Image Pro Plus software. E) Representative images of spheroid sprouting angiogenesis assay at 12 and 24 h after cell spheroids were seeded on Type I collagen. EC‐CM(BMSCs) group: hUVECs were cultured in a conditioned medium derived from hBMSCs; EC‐CM(BMSCs^mito^) group: hUVECs were cultured in a conditioned medium derived from hBMSCs^mito^. Scale bar, 100 µm. F) The migration distance of each group in Figure E was quantified using the Image Pro Plus software. G) Representative images of tube formation at 4 and 8 h after cells were seeded on Matrigel. EC (+BMSCs) group: hUVECs were transwell cultured with hBMSCs; EC (+BMSCs^mito^) group: hUVECs were transwell cultured with hBMSCs^mito^. Scale bar, 100 µm. (H) The total number of tubules, number of junctions, and tube lengths of each group in Figure G were quantified using the Image Pro Plus software.

In our investigation of the underlying mechanisms of how pro‐angiogenic functions are enhanced by mitochondrial transplantation, we explored its impact in both indirect and direct co‐culture experiments involving BMSCs^mito^ and ECs. BMSCs^mito^, labeled with green fluorescence, and hUVECs, labeled with red fluorescence, facilitated in vitro tube formation and spheroid sprouting. Confocal microscopy revealed intimate and uniform contact between these cell types (**Figure** **4**A,B), thus indicating the key roles of the transplanted mitochondria in promoting a more cohesive distribution and a larger area of mutual interaction. Physiologically, the proximity of MSCs and ECs is recognized to foster angiogenesis,^[^ ^18^ ^]^ with MSCs and ECs engaging in complex interactions in vivo.^[^ ^5^, ^19^ ^]^ These findings thus suggest that an essential mode of cell communication occurs through direct cell‐cell interactions.

By contrast, indirect co‐culture involving BMSCs^mito^ showed no significant difference in promoting angiogenesis when compared to the control group with BMSCs (Figure 4C–H). This suggests that cell communication may not involve mechanisms beyond soluble factor secretion. Therefore, we adopted a direct co‐culture model, which demonstrated a marked enhancement of angiogenesis driven by direct cell‐cell interaction.

### 2.4 Dll4‐Notch1 Signaling is Essential for BMSCs^mito^ to Promote Endothelial Tube Formation

The Notch signaling pathway plays a fundamental role in direct cell‐to‐cell interactions, particularly within the skeletal system, thereby exerting a profound influence on bone angiogenesis and osteogenesis.^[^ ^19^ ^]^ Hence, we aimed to elucidate the crucial role of BMSCs^mito^ in promoting endothelial tube formation by exploring its association with the Notch signaling pathway. To delve into the underlying downstream mechanisms and their potential connection with the Notch signaling pathway, we engaged in a 24‐hour direct co‐culture of BMSCs^mito^ and ECs, followed by flow cytometry‐based sorting for the isolation of pure populations of BMSCs^mito^ and ECs.

**Figure 5:**
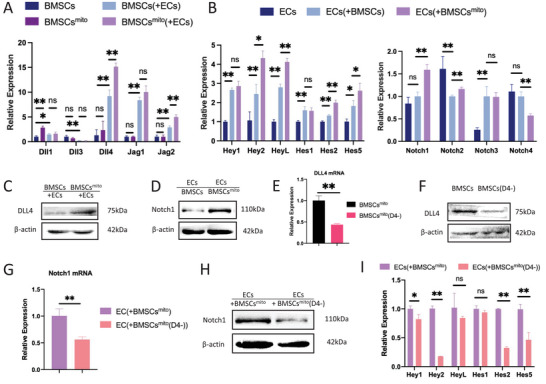
Activation of DLL4‐Notch signaling in the BMSCs^mito^‐ECs co‐culture system. A) The mRNA expression levels of the Notch signaling pathway ligand of BMSCs^mito^. B) The mRNA expression levels of the Notch signaling pathway downstream molecules and receptors in hUVECs after co‐culture. C,D) Protein expression levels of DLL4 in BMSCs^mito^ and Notch1 in hUVECs after co‐culture, β‐actin was utilized as the internal control. E,F) qPCR and western blot analyses of DLL4 expression in of hBMSCs after transfection with lentivirus for down‐regulating expression of DLL4, with β‐actin being used as the internal control. G,H) mRNA and protein expression levels of Notch1 receptor in hUVECs co‐cultured with BMSCs^mito^ after down‐regulating expression of DLL4. I) mRNA expression levels of Notch signaling pathway downstream molecules in hUVECs co‐cultured with BMSCs^mito^ after down‐regulating expression of DLL4. Results are presented as the mean ± SEM (*n*  =  3). One‐way ANOVA with Tukey's post hoc test was used to determine statistical significance. *p < 0.05, **p < 0.01, ns: no significant difference.

Co‐culturing BMSCs^mito^ with ECs revealed a significant increase in both DLL4 mRNA and protein expression levels compared to control‐BMSCs (**Figure** **5**A,C). Additionally, co‐culturing ECs with BMSCs^mito^ led to enhanced activation of downstream signaling molecules within the Notch1 receptor and corresponding Notch signaling pathways (Figure 5B,D).

**Figure 6:**
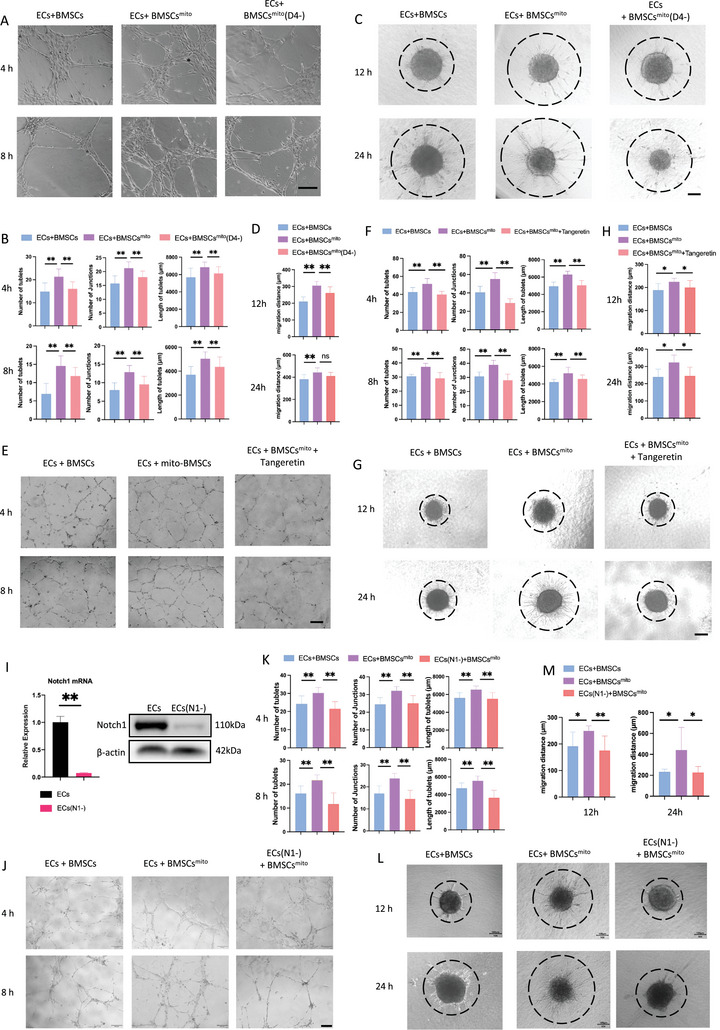
Effects of dll4‐Notch1 signaling on angiogenesis in the BMSCs^mito^‐ECs co‐culture system. A,E,J) Representative images of tube formation at 4 and 8 h after cells were seeded on Matrigel and C,G,L) Representative images of spheroid sprouting angiogenesis assays at 12 and 24 h after cell spheroids were seeded on Type I collagen. ECs + BMSCs group: hUVECs co‐culture with hBMSCs at a ratio of 4:1. ECs + BMSCs^mito^: hUVECs co‐culture with BMSCs^mito^ at a ratio of 4:1; ECs + BMSCs^mito^ (D4‐) group: hUVECs co‐culture with BMSCs^mito^ after transfection with lentivirus for down‐regulating expression of DLL4 at a ratio of 4:1; ECs + BMSCs^mito^ + Tangeretin group: hUVECs co‐culture with BMSCs^mito^ at a ratio of 4:1 in culture medium containing 1% (w/v) Tangeretin; ECs(N1‐) + BMSCs^mito^ group: hUVECs after transfection with lentivirus for down‐regulating expression of Notch1 co‐culture with BMSCs^mito^ at a ratio of 4:1. Scale bar, 100 µm. B) The total number of tubules, number of junctions, and tube lengths in each group in Figure A were quantified using the Image Pro Plus software. D) The migration distance of each group in Figure C was quantified using the Image Pro Plus software. F) The total numbers of tubules, number of junctions, and tube lengths of each group in Figure E were quantified using the Image Pro Plus software. H) The migration distances of each group in Figure G were quantified using the Image Pro Plus software. I) qPCR and western blot analyses of Notch1 expression in hUVECs after transfection with lentivirus for down‐regulating expression of Notch1, with β‐actin utilized as the internal control. K) The total number of tubules, number of junctions, and tube lengths of each group in Figure J were quantified using the Image Pro Plus software. M) The migration distances of each group in Figure L were quantified using the Image Pro Plus software. Results are presented as the mean ± SEM (*n*  =  3). One‐way ANOVA with Tukey's post hoc test was used to assess statistically significant differences. *p < 0.05, **p < 0.01, ns: no significant difference.

To validate the role of the DLL4‐Notch1 signaling pathway, we inhibited DLL4 in hBMSCs [BMSCs^mito^ (D4‐)] (Figure 5E,F). In parallel, we induced Notch1 knockout in ECs [ECs (N1‐)] (Figure G,H) and utilized the Notch1 inhibitor, Tangeretin. In vitro tube formation assays exhibited reduced angiogenic functionality in the BMSCs^mito^ (D4‐) ‐ECs group compared to the BMSCs^mito^‐ECs group (**Figure** **6**A–D). Moreover, co‐culturing ECs with BMSCs^mito^ (D4‐) led to decreased expression levels of Notch1 and downstream signaling genes in contrast to co‐culture with BMSCs^mito^ (Figure 5I). Extending these observations, similar reductions in angiogenic functionality were observed upon viral vector or inhibitor‐induced suppression of Notch receptors or signaling pathways in ECs (Figure 6E–L). These data thus underscore the key involvement of the DLL4‐Notch1 signaling pathway in the pro‐angiogenic effects of BMSCs^mito^.

### 2.5 BMSCs^mito^ Enhanced Functional Vascular Network Formation and Therapeutic Effects on Bone Repair Upon Co‐Transplantation with ECs

**Figure 7:**
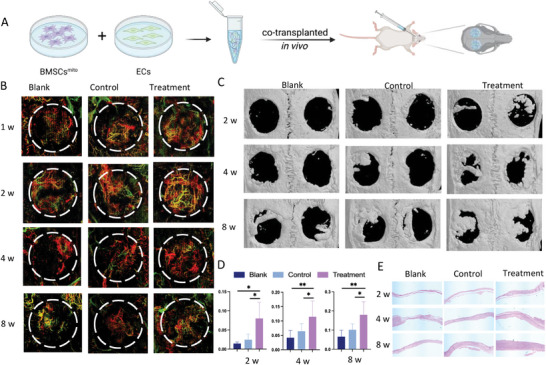
BMSCs^mito^ enhanced the functional vascular network formation and therapeutic effects on bone repair upon co‐transplantation with ECs. A) Illustration of the co‐transplantation procedure in a rat cranial defect model. B) Representative imaging of blood vessels in a rat cranial critical‐sized full‐thickness defects at 1, 2, 4, and 8 weeks after surgery. The dashed line demarcated the bone defect area. Blood vessel depth was color‐coded: green indicated shallow vessels, while red indicated deep vessels. C) Representative micro‐CT images of rat cranial critical‐sized full‐thickness defects at 2, 4, and 8 weeks after surgery. D) Quantitative analysis of bone mineral density (BMD) of the newly‐formed bone. E) Scans of HE staining of bone tissue slices of specimens. Scale bar, 200 µm. Results are presented as the mean ± SEM (*n * =  5). One‐way ANOVA with Tukey's post hoc test was used to determine statistical significance. *p < 0.05, **p < 0.01, ns: no significant difference.

Based on the positive outcomes observed with BMSCs^mito^‐ECs in promoting angiogenesis both in vivo and in vitro, our study subsequently delved into the assessment of three distinct groups: the blank group, control groups (BMSCs‐ECs), and treatment groups (BMSCs^mito^‐ECs). The primary focus was to scrutinize the development of functional vascular networks and evaluate the progression of bone repair within these experimental groups, using a rat calvarial defect model (**Figure** **7**A).

Optical Coherence Tomography Angiography imaging detected the blood flow signals within the tissue defect, outlining the vascular structures. The OCTA results at 1 and 2 weeks post‐surgery revealed a significantly higher density of robust blood vessel formation in the experimental group compared to both the control and blank groups (Figure 7B). Additionally, micro‐CT images captured at 2‐, 4‐, and 8 weeks post‐surgery revealed a greater accumulation of newly formed bone in the experimental group versus the control and blank groups (Figure 7C). Bone mineral density (BMD) analysis indicated a significantly higher BMD value in the experimental group versus the control group (Figure 7D,E). Histological assessments depicted the presence of newly formed bone within the defect area of the experimental group (Figure 7E).

These collective findings thus underscored the remarkable potential of mitochondrial transplantation in promoting BMSCs^mito^‐ECs signaling crosstalk that enhanced both angiogenesis and bone regeneration. Mitochondrial transplantation has previously been explored in various therapeutic contexts, including bone regeneration. However, our study advances this field by demonstrating that the transplant of healthy mitochondria into hBMSCs and establishing an optimized BMSCs^mito^‐ECs co‐culture system significantly enhances their angiogenic capacity in both in vitro and in vivo models.

It is worth mentioning that native MSCs, particularly those residing within tissues, often act as perivascular cells. As Crisan et al.^[^ ^20^ ^]^ demonstrated, that these cells actively support and maintain vascular integrity within the vascular niche. This aligns with the ability of BMSCs^mito^ to reassociate with ECs as pericytes when co‐cultured. The role of DLL4‐Notch signaling in pericyte formation during vasculogenesis, as elucidated by Schadler‐Stewart et al.,^[^ ^21^ ^]^ provides mechanistic insight into our observations. Our study found that the DLL4‐Notch1 signaling pathway is crucial for the enhanced angiogenic activity observed in BMSCs^mito^‐EC co‐cultures. This suggests that mitochondrial transplantation may enhance the intrinsic pro‐angiogenic functions of MSCs by boosting their pericyte‐like properties.

In bone regeneration, the spatial arrangement and interaction of cells within a 3D matrix are crucial for effective tissue formation and vascularization.^[^ ^22^ ^]^ For instance, BMSC‐EC co‐cultures in 3D environments, particularly when supported by extracellular matrix (ECM) components, have demonstrated substantial improvements in angiogenic outcomes.^[^ ^23^ ^]^ Studies have shown that 3D aggregates of BMSCs and ECs, bolstered by ECM components, can create a more physiologically relevant environment that supports robust angiogenesis.^[^ ^24^ ^]^ Since 3D aggregates bolstered by ECM may effectively improve angiogenesis and perhaps, this might also synergize with mitochondrial transplantation. These 3D configurations may also facilitate basal mitochondrial transfer between cells, enhancing their overall regenerative potential.

This research thus suggested that BMSCs receiving mitochondrial transplantation promoted the angiogenesis and bone regeneration of ECs, which might provide an innovative and feasible approach for therapy in bone tissue regeneration. However, considering the risks of tumor formation by of mesenchymal stem cells, there is still a long way to go before mitochondria transplantation of BMSCs can be realized in a therapeutic setting.

## 3 Conclusion

In this study, we utilized mitochondrial transplantation technology to design an optimized BMSCs^mito^‐ECs co‐culture system, emphasizing its enhanced capabilities in promoting angiogenesis and bone regeneration. We found that BMSCs^mito^‐ECs significantly amplified angiogenic functions both in vitro and in vivo, additionally demonstrating a substantial improvement in vascular network formation and bone repair efficacy within a rat cranial bone defect model. This study thus highlighted the key roles of direct cell‐cell interactions and the Dll4‐Notch1 signaling pathway in these processes. The results underscore the potential of mitochondria transplantation as a promising strategy to boost angiogenesis and osteogenesis during bone repair, thus offering a potent tool for improving treatment outcomes in regenerative medicine.

## 4 Experimental Section

### Cell Culture

The two human primary cell cultures, hUVECs, and hBSMCs, were purchased from ScienCell Research Laboratories and were cultured in commercially available mesenchymal stem cell medium (MSCM; 7501; ScienCell Research Laboratories, Carlsbad, CA USA) and endothelial cell medium (ECM; 1001; ScienCell Research Laboratories). The two primary Sprague‐Dawley rat cell cultures, rBMSCs and RAECs, were purchased from Procell Life Science&Technology Co., Ltd. (Wuhan, China) and cultured in α‐MEM (Hyclone SH30265.01) and DMEM with high glucose (Hyclone SH300222.01) supplemented with 10% (v/v) fetal bovine serum (FBS) (Procell 164210–50) and 1% (v/v) penicillin‐streptomycin solution (Procell PB180120). Cells were cultured in a humidified incubator with 5% CO_2_ at 37 °C. The culture medium was refreshed every 1 to 2 days. All cells utilized in experiments were between passages 4–7. Cellular and mitochondrial exposures to EDTA were avoided at all steps in the experiments. For mitochondria transplantation, both donor and recipient BMSCs were seeded into a 6‐well plate at 2 × 10^5^ cells per well, with donor BMSCs being harvested after 36 h. The Mitochondria Isolation Kit for Cultured Cells (ThermoFisher, Rockford, Illinois, USA) was utilized to isolate mitochondria from donor BMSCs according to the manufacturer's instructions. A series of differential centrifugation steps were carried out to separate the mitochondrial and cytosolic fractions. The isolated mitochondria were directly resuspended in 1 mL of complete medium and kept on ice before transplantation. The supernatant of the recipient BMSCs was removed, and the mitochondria suspension was added slowly close to the bottom of the well. As for the control BMSCs, the supernatant was also removed, and 1 mL of medium without mitochondria was added instead. The whole plate was centrifuged at 1500 rcf at 4 °C for 15 min, placed within a 37 °C incubator for 2 h, and centrifuged under the same conditions again, to facilitate cellular mitochondria uptake. The cells were then placed back into a 37 °C incubator for 24 h before subsequent experiments.

### In Vitro Tube Formation Assays

Under sterile conditions, the 24‐well plates were coated with 250 µL Matrigel (Becton, Dickinson and Company, Franklin Lakes, NJ, USA) per well without air bubbles. The plates were then incubated at 37 °C for at least 30 min to allow the Matrigel to set. Next, hUVECs: hBMSCs at a ratio of 16:1 (11.3 × 10^4^ of hUVECs and 0.7× 10^4^ of hBMSCs), hUVECs: hBMSCs at a ratio of 8:1 (10.7 × 10^4^ of hUVECs and 1.3 × 10^4^ of hBMSCs), hUVECs: hBMSCs at a ratio of 4:1 (9.6 × 10^4^ of hUVECs and 2.4 × 10^4^ of hBMSCs), hUVECs: hBMSCs at a ratio of 1:1 (6 × 10^4^ of hUVECs and 6 × 10^4^ of hBMSCs), and hUVECs (12 × 10^4^ of hUVECs) were plated on the Matrigel. Finally, 300 µL of medium was added, and the 24‐well plates were incubated at 37 °C within a 5% CO_2_ incubator. The formation of tube structures was examined under a phase contrast microscope after 4 and 8 h.

### Generation of Endothelial Cell Spheroids

Agarose at (2% w/v) was used to form a mold for cell spheroids, whilst preventing the adhesion of cells to the mold surface. The agarose was heated to form a melted solution and then added to a 3D Petri Dish (Microtissues). After solidification, the agarose molds were placed in a six‐well plate for culturing cells. Then, 180 µL of cell suspension were constituted with hUVECs: hBMSCs at a ratio of 4:1 (12 × 10^5^ of hUVECs and 3 × 10^5^ of hBMSCs), hUVECs: hBMSCs at a ratio of 8:1 (13.3 × 10^5^ of hUVECs and 1.7 × 10^5^ of hBMSCs) and hUVECs (15 × 10^5^ of hUVECs) were seeded on each mold. Twenty minutes later, the culture medium was added and cellular aggregates were allowed to form for 24 h.

### The Spheroid‐Based Sprouting Angiogenesis Assay

For the in vitro sprouting angiogenesis assay, the spheroids were generated overnight, after which they were embedded into Collagen Type I (Corning, USA) with medium added, and the plates were incubated at 37 °C to allow the Collagen to gel. The spheroids were then allowed to sprout for 12 and 24 h. Then, in vitro sprouting was quantitated digitally by measuring the length of the spouts that had grown out of each spheroid using the Image Pro Plus software, and by analyzing 8 spheroids per experimental group.

### Lentivirus Production and Transfection

All lentivirus vectors fused with green fluorescent protein (GFP) for knockdown of Notch1 and DLL4 were purchased from Shanghai Genechem Co., Ltd. (Shanghai, China).

Cells transfected with scramble were assigned as controls. One day before lentiviral transfection, hBMSCs and hUVECs were seeded in a 6‐well plate at a density of 15 000 cells cm^−2^. Next, 1 µL of lentiviral particles (1.5 × 10^8^TU mL^−1^; Shanghai Genechem Co., Ltd, Shanghai, China) were added with 5 µg mL^−1^ polybrene (Shanghai Genechem Co., Ltd) and 1 mL of medium to the cell culture for 16 h. Next, the transfected cells were selected using puromycin (P8833; Sigma‐Aldrich) for 3 days.

### Western Blot

The cultured cells were lysed with RIPA lysis buffer (Beyotime, Shanghai, China) supplemented with a protease inhibitor cocktail (ThermoFisher, Rockford, Illinois, USA) on ice. The protein concentration was quantified using a BCA protein assay kit (ThermoFisher, Rockford, Illinois, USA). Six times concentrated SDS Sample Loading Buffer (P0015F; Beyotime) was added to the protein before heating at 100 °C for 5 min. Then, the total protein extract (30 µg) was separated by 10% (w/v) sodium dodecyl sulfate polyacrylamide gel electrophoresis, and the proteins were transferred to a PVDF membrane. The membranes were blocked with 5% (w/v) skimmed milk and then incubated with the primary antibody at 4 °C overnight, followed by incubation with a secondary antibody conjugated with horseradish peroxidase (HRP). Autoradiography was performed with an eECL Western Blot Kit (CoWin Bio., Jiangsu, China) on a film exposure machine. The primary antibodies Notch1(A19090) and DLL4(A12943) were purchased from Abclonal. The primary antibody against β‐Actin (AF0003) and secondary antibody HRP‐labeled IgG (A0208, A0216) was purchased from Beyotime, China. β‐Actin was utilized as the protein loading control. The protein expression levels were normalized to β‐Actin.

### Real‐Time Quantitative RT‐PCR Analysis

Total RNA extraction was carried out using TRIzol Reagent (Invitrogen, USA) according to the manufacturer's instructions. Amplifications were then performed with the different primers. The quality and quantity of the RNA obtained were subjected to spectrophotometric analysis using a bio‐photometer (Thermo Scientific NanoDrop8000). The RNA was then reversed‐transcribed into complementary DNA (cDNA) using a Reverse Transcription kit (Takara Bio Inc., Japan). Quantitative real‐time polymerase chain reaction (qPCR) was performed with the SYBR Green PCR reagent kit (Roche, Germany) on an ABI QuantStudio 3 Real‐Time PCR System (Applied Biosystems, Foster City, CA, USA). All values were normalized to GAPDH.

### Animal Experiments: Animals and Surgical Procedures

A total of sixty 7‐week‐old male Sprague‐Dawley (SD) rats were used in this study. The experimental protocol was approved by the Animal Care and Use Committee of Peking University (LA2017108 and LA2019297). To establish the cranial defect model, the dorsal cranium was surgically exposed after the rats were anesthetized by phenobarbital sodium (100 mg kg^−1^) via intraperitoneal injections. Two critical‐sized full‐thickness bone defects (5 mm in diameter) on each side of the parietal bone were created by a saline‐cooled trephine drill. There were 3 groups (*n* = 5): blank—both sides filled with Matrigel only; control—both sides filled with Matrigel and 4 × 10^5^ rAECs and 1 × 10^5^ control rBMSCs; and treatment—both sides filled with Matrigel and 4 × 10^5^ rAECs and 1 × 10^5^ rBMSCs^mito^ for each defect.

Eighteen 8‐week‐old male Balb/c nude mice with cells injected subcutaneously were used in this study. There were 3 groups (*n* = 6): blank—both sides injected with Matrigel and 10 × 10^6^ hUVECs; control—both sides injected with Matrigel and 8 × 10^6^ hUVECs and 2 × 10^6^ control hBMSCs; and treatment—both sides injected with Matrigel together with 8 × 10^6^ hUVECs and 2 × 10^6^ hBMSCs^mito^ for each side. After 2 and 4 days, the transplants were harvested.

### Animal Experiments: Micro‐CT Scanning Evaluation

At 1‐, 2‐, 4‐, and 8 weeks post‐transplantation, calvaria samples were harvested and fixed in 4% (w/v) paraformaldehyde for 36 h at 4 °C. The specimens were then examined using a Viva40 micro‐CT scanner (Scanco Medical. AG). The bone volume was analyzed, and 3D reconstruction was carried out based on the processed images using Scanco software.

### Animal Experiments: Histological Analysis

Following micro‐CT analysis, rat skulls were decalcified and paraffin‐embedded. Histomorphology analysis was performed on 5‐µm‐thick histology sections of the central portions of the skull defects. The sections were then subjected to hematoxylin and eosin (H&E) and Masson's trichrome staining, according to the manufacturer's protocols. Images were captured using an Olympus D70 camera mounted on a Nikon Eclipse E800 microscope.

### Animal Experiments: Statistical Analysis

Results were expressed as mean ± SEM. Analysis between more than two groups was performed by a one‐way unstacked ANOVA and post‐hoc LSD testing. Analysis between two paired samples was performed by a two‐tailed unpaired Student's *t*‐test. *p* values of less than 0.05 were considered to be statistically significant.

## Conflict of Interest

The authors declare no conflict of interest.

## Supporting information

Supporting Information

## Data Availability

The data that support the findings of this study are available in the supplementary material of this article.
