# Supplementary material for: Mitochondria Transplantation to Bone Marrow Stromal Cells Promotes Angiogenesis During Bone Repair
Source: Adv Sci (Weinh). 2024 Aug 13;11(39):2403201. doi: 10.1002/advs.202403201 (PMC11497025; doi:10.1002/advs.202403201)
Supplement: Supplementary file 1 — Supporting Information [file ADVS-11-2403201-s001.docx]

**Supporting information figures**


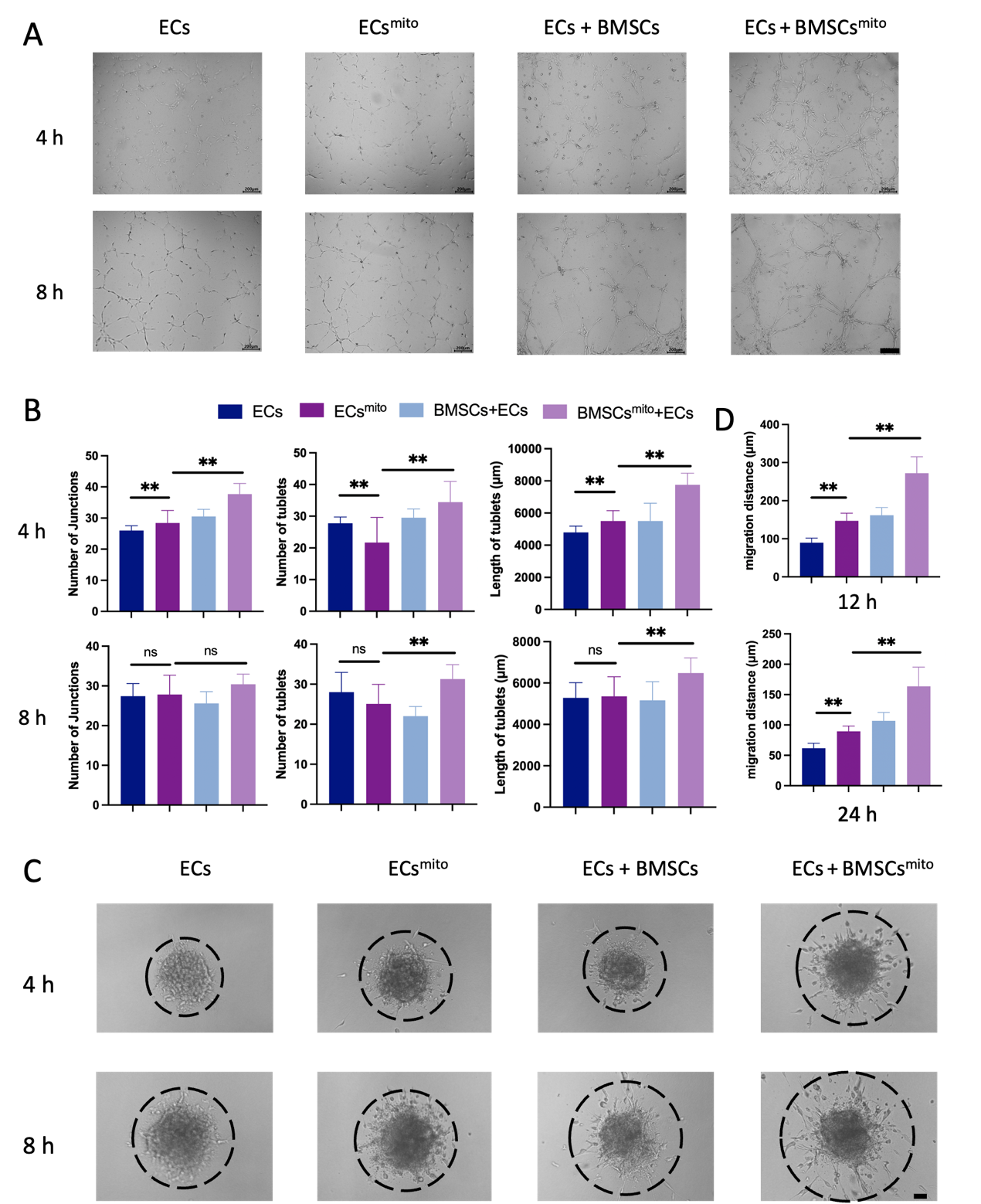


**Figure S1 Mitochondria enhance the angiogenic function of BMSCs, surpassing direct promotion of vascular functionality in ECs** (A) Representative images of tube formation at 4 and 8 h after cells were seeded on Matrigel. ECs group: independent culture of ECs. ECs^mito^ group: independent culture of ECs after mitochondria transplantation. ECs + BMSCs group: hUVECs co-culture with hBMSCs in a ratio of 4:1. ECs + hBMSCs^mito^ : hUVECs co-culture with BMSCs^mito^ in a ratio of 4:1. Scale bar, 200μm. (B) The total number of tubules, number of junctions, and tube length in each group in (A) were quantified using Image Pro Plus software. (C)Representative images of spheroid sprouting angiogenesis assay at 12 and 24 h after cells spheroid were seeded on Type I collagen. ECs group: independent culture of ECs. ECs^mito^ group: independent culture of ECs after mitochondria transfer. ECs + BMSCs group: hUVECs co-culture with hBMSCs in a ratio of 4:1. ECs + BMSCs^mito^: hUVECs co-culture with hBMSCs^mito^ in a ratio of 4:1. Scale bar, 100μm. (D) The migration distance in each group in Figure C were quantified using Image Pro Plus software.


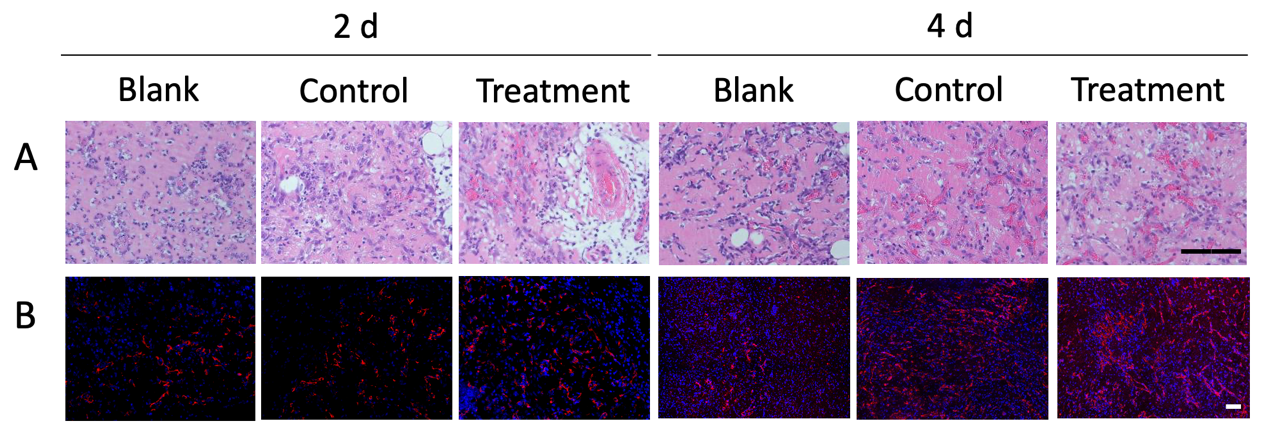


**Figure S2 Co-transplanted of BMSCsmito and hUVECs increased vascularization in vivo**

Blank group: independent inject of hUVECs. Control group: hUVECs and hBMSCs were co-cultured in a ratio of 4 : 1; Treatment group: hUVECs and BMSCsmito were co-cultured in a ratio of 4 : 1.

Representative images depict high-magnification HE staining (A) and show low-magnification CD31 staining (B) of implants tissue slices of specimens. Scale bar, 50μm.
